# The Derby Infirmary

**Published:** 1890-06-28

**Authors:** 


### Editor's Letter-Box.

[Our Correspondents are reminded that prolixity is a great bar to publication, and that brevity of style and conciseness of statement greatly facilitate early insertion.]

### THE DERBY INFIRMARY.

Dr. C. H. Taylor, House Surgeon at the Derby Infirmary, writes: Had you applied to me for information about the sanitary defects of this hospital, I should have been very pleased to have furnished you with the same, and so saved you from some errors. Neither doctors nor nurses have fled. As our in-patient department has been reduced from 175 beds to 75, several nurses for whom there was no employment, have been drafted elsewhere. The statement about reconstruction costing £50,000 is quite beside the mark. Reconstruction has not been discussed definitely yet. That sum was mentioned as an approximate amount for *rebuilding* all the defective blocks. One large block requiring alteration only, not rebuilding. Mr. Rogers Field, the Sanitary Engineer, has already examined and reported on the whole sanitary condition, and has practically endorsed Messrs. Young and Hall's report. Our Committee fully realize what has to be done, but feel how difficult it is to make the public understand that a building which outwardly presents so imposing an appearance, can be so radically defective, that nothing short of "uprooting" it will suffice. With a view to strengthening their case they are about to ask Sir Douglas Galton to come and advise them on the whole question.

